# Supplementary material for: The origin and evolution of IncF33 plasmids based on large-scale data sets
Source: mSystems. 2023 Sep 26;8(5):e00508-23. doi: 10.1128/msystems.00508-23 (PMC10654068; doi:10.1128/msystems.00508-23)
Supplement: Legends — to Fig. S1 to S4. [file msystems.00508-23-s0005.docx]

**FIG S1** Antibiotic resistance genes carried by IncF33 plasmids. (a) Distribution of major antibiotic resistance genes in IncF33 plasmids. (b) The percentage of IncF33 plasmids carrying different numbers of resistance genes.

**FIG S2** Linear comparison of IncF33 plasmids lacking some backbone fragments (1-5). Over 99% homologous segments are indicated by grey shading. Genes are represented by arrows and are classified by function into different groups.

**FIG S3** Linear comparison of IncF33 plasmids lacking some backbone fragments (6-13). Over 99% homologous segments are indicated by grey shading. Genes are represented by arrows and are classified by function into different groups.

**FIG S4** Linear comparisons of IncF33 plasmids lacking some backbone fragments (14-16). Over 99% homologous segments are indicated by grey shading. Genes are represented by arrows and are classified by function into different groups.
