# Supplementary material for: The origin and evolution of IncF33 plasmids based on large-scale data sets
Source: mSystems. 2023 Sep 26;8(5):e00508-23. doi: 10.1128/msystems.00508-23 (PMC10654068; doi:10.1128/msystems.00508-23)
Supplement: Table S4 — Information on IncF33 cointegrate plasmids. [file msystems.00508-23-s0007.docx]

**Tables S4. Information on IncF33 cointegrate plasmids**

| **Plasmid types (Participating in the formation of cointegrates)** | **Cointegrates (Type)** | **Components of cointegrate plasmid** | **Host range (Replication modules other than IncF33)** |
| --- | --- | --- | --- |
| IncR | IncR-F33:A-:B- (Type III) | Complete IncR plasmids and incomplete IncF33 plasmids (missing most of the conjugative transfer region genes) | Broad (IncR) |
| IncN1, rolling-circle plasmid | IncN1-F33:A-:B-(Type I) | Incomplete IncN plasmids (missing almost all of the conjugative transfer region genes), incomplete IncF33 plasmids (Containing only replication region and variable region genes) and incomplete rolling-circle plasmid (Containing replication protein) | Broad (IncN), Unknown(rolling-circle plasmid) |
| IncX1 | IncX1-F33:A-:B-(Type II) | IncX1 plasmid (lacking partial tra genes) and complete IncF33 plasmid | Narrow (IncX1) |
| IncN1 | IncN1-F33:A-:B-(Type I) | IncN1 plasmid fragment (replication region genes) and complete IncF33 plasmid | Broad (IncN) |
| IncX1 | IncX1-F33:A-:B-(Type I) | IncX1 plasmid fragment (replication region and addiction system genes) and complete IncF33 plasmid | Narrow (IncX1) |
| p0111 (phage-like plasmids), IncN1 | IncN1-p0111-F33:A-:B- (Type II & Type I) | Complete p0111 plasmids, IncF33 plasmids and IncN1 plasmid fragment (replication region genes) | Broad (IncN), Broad(p0111) |
| IncFIA | IncFIA-IncN1-F33:A-:B- (Type II) | Novel IncFIA-FIB plasmid (lacking partial tra genes) , complete IncF33 plasmid and IncN plasmid fragment (replication region genes) | Narrow(IncFIA), Broad (IncN) |

**Reference**

Liu YY, et al. 2021. The formation of two hybrid plasmids mediated by IS*2*6 and Tn*6952* in *Salmonella enterica* serotype enteritidis. Frontiers in Microbiology 12:676574. doi: 10.3389/fmicb.2021.676574.

He DD, et al. 2019. Emergence of a hybrid plasmid derived from IncN1-F33:A-:B- and *mcr-1*-bearing plasmids mediated by IS*26*. Journal of Antimicrobial Chemotherapy 74 (11):3184-3189. doi: 10.1093/jac/dkz327.

Hua XT, et al. 2020. Cointegration as a mechanism for the evolution of a KPC-producing multidrug resistance plasmid in *Proteus mirabilis*. Emerg Microbes Infect 9 (1):1206-1218. doi: 10.1080/22221751.2020.1773322.

Xiang DR, et al. 2016. Complete sequence of a novel IncR-F33:A-:B- plasmid, pKP1034, harboring *fosA3*, *bla*_KPC-2_, *bl*a_CTX-M-65_, *bla*_SHV-12_, and *rmtB* from an epidemic *Klebsiella pneumoniae* sequence type 11 strain in China. Antimicrobial Agents Chemotherapy 60 (3):1343-1348. doi: 10.1128/AAC.01488-15.
